# Vigilant Fiberoptic Orotracheal Intubation in a Patient With Severe Craniofacial Trauma During the Last Morocco Earthquake

**DOI:** 10.7759/cureus.67746

**Published:** 2024-08-25

**Authors:** Mehdi Nabil, Said Khallikane, Abdeljalil Abouchadi, Issam Serghini, Qamouss Youssef

**Affiliations:** 1 Anesthesiology and Reanimation, Military Hopital of Avicenne, Marrakech, MAR; 2 Anesthesiology and Critical Care, Military Hopital of Avicenne, Marrakech, MAR; 3 Maxillofacial Surgery, Military Hopital of Avicenne, Marrakech, MAR; 4 Emergency Service, Military Hopital of Avicenne, Marrakech, MAR; 5 Anesthesia and Critical Care, Cadi Ayyad University, Military Hopital of Avicenne, Marrakech, MAR

**Keywords:** severe, craniofacial trauma, fiberoptic, orotracheal intubation, vigilant

## Abstract

Maxillofacial trauma is prevalent, particularly among the young population, often stemming from assaults, road accidents, or sports-related mishaps. Traditional intubation methods for managing these injuries can be challenging, especially with occluso-facial fractures requiring intermaxillary blocking for dental articulation restoration. Effective management requires interdisciplinary collaboration between emergency physicians, anesthetists, and maxillofacial surgeons. Proficiency in techniques like the vigilant fiberoptic approach should be emphasized through specialized training courses. This collaborative approach ensures the best possible strategy for managing difficult airways, with input from all stakeholders including patients, students, and practitioners. In this case, we successfully conducted a rapid-sequence awake fiberoptic oral intubation on a trauma patient, during the last earthquake that hit Morocco, with severe craniofacial injuries and an unstable skull. The patient, a 40-year-old woman, presented with complex facial fractures, including hemi lefort III on the right and hemi lefort II on the left, along with minimal subarachnoid hemorrhage and frontal pneumocephalus. Due to the patient's compromised airway from diffuse facial bleeding and low oxygen saturation, we opted for awake fiberoptic intubation once immediate life-threatening issues were addressed. This approach allowed us to maintain the patient's spontaneous respirations and navigate around unstable craniofacial structures. The procedure was performed with meticulous care, considering the patient's unstable skull, and was successful without complications. Post-intubation, the patient was extubated, and her recovery was uneventful.

## Introduction

Maxillofacial trauma is common, mainly affecting a young population. These traumas are often caused by attacks, road accidents, and sporting accidents. Conventional intubation techniques can be challenging for surgical management. Dental articulation restoration is achieved with intermaxillary blocking [1]. It is clear that communication challenges hinder finding precise solutions across fields and among experts. We propose emergency physicians learn the vigilant fiberoptic approach, collaborating with anesthetists and maxillofacial surgeons for managing difficult airways. Severe craniofacial trauma incidence has decreased with helmet use, but patients with varying severity still exist. Wired fiberoptic endoscopes have improved intubation, allowing for rapid, simpler procedures without premedication. Transitioning to oral intubation necessitates conscious control techniques and topical anesthesia [2]. In this high-risk patient profile, WiredScopel Inc. introduced "The Microlaryngoscope", a revolutionary device with a fiberoptic and suction channel, and high-resolution videos for pulmonologists. Positive comments were received from anesthetists. However, no published management has been experienced with this device in trauma patients. Virtual fiberoptic has been successful in difficult intubation cases. Airway management in severe craniofacial trauma patients is challenging. Premedicants may be given to these patients but can cause hypotension, hypoxia, and hypoventilation when combined with local anesthetic [2-4]. As with urgent resuscitative decisions, both consensus and personal practice are essential. Indeed, even longstanding consensus is revisited after due consideration over the past year. Applications of monitoring, intubating techniques/materials, and definitive airway options continue to change. Along with these considerations, initial choices of airway readiness or drug-assisted intubation options are determined in every patient's room. Presently, a "delicate" approach for most patients is considered: minimizer techniques for pulmonary toilet, pharmacologic direct or video laryngoscopy, and delayed awake intubation protocols in the well-prepared patient with the use of the monitor [5-7]. The decisions for urgent tracheal intubation include craniofacial fractures, incomplete neurologic examination, ventilatory or circulatory collapse, Glasgow Coma Scale (GCS) <9, flail chest, pneumonia, or compromised airway due to secretions. Temporizing aggressive airway management is also decided for patients with GCS <9 to allow for intracranial intervention. Assessment of facial fractures guides early airway management, considering factors such as fracture site, number, displacement, soft tissue injuries, and medical issues [6].

## Case presentation

**Figure 1:**
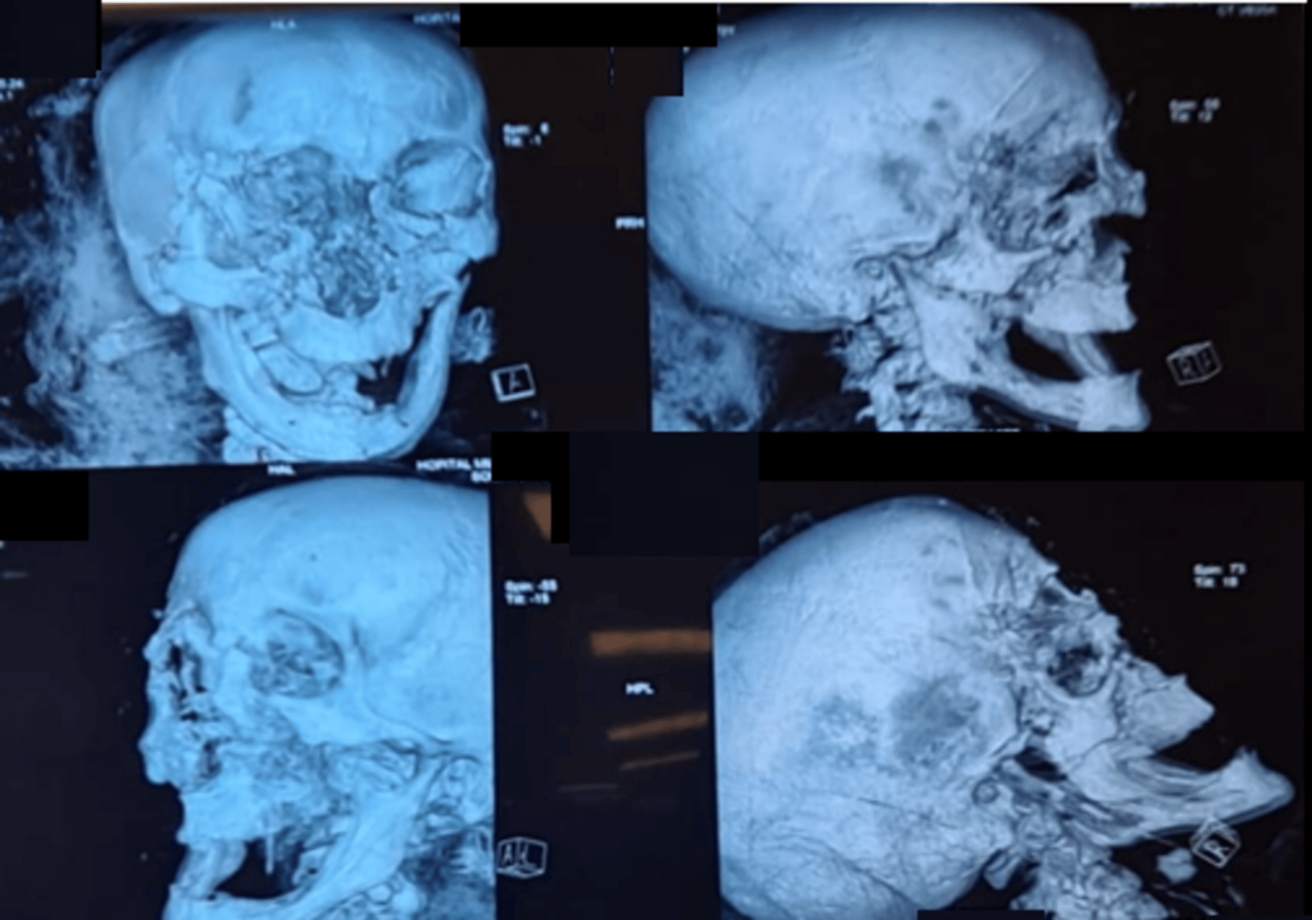
CT scan in 3D reconstruction revealing multiple facial fractures.

We report the case of a successful awake fiberoptic orotracheal intubation in a trauma patient with severe craniofacial trauma and an unstable skull, which occurred during the last earthquake that hit Morocco. The patient was a 40-year-old woman who presented with craniofacial injuries. As part of the injury assessment, a body scan was carried out, showing minimal subarachnoid hemorrhage, compressive frontal pneumocephalus (Mont Fuji) with complex facial fractures, hemi lefort III on the right, and hemi lefort II on the left (Figure 1).

**Figure 2:**
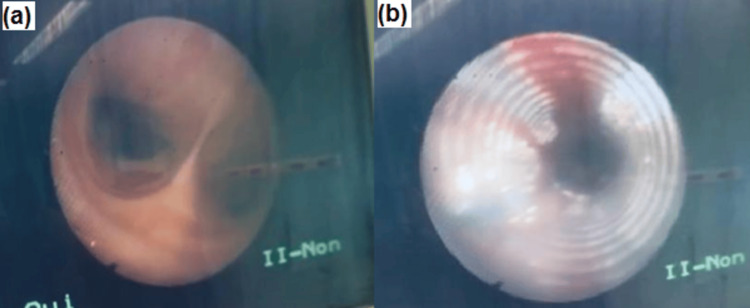
View of the carina (a) and endotracheal tube (b) during orotracheal intubation using bronchoscopic guidance.

Despite maintaining spontaneous respirations, the patient was in a semiconscious mental status of Glasgow Coma Scale (GCS) of 13, and her airway was restricted due to diffuse bleeding in various facial sites and associated low oxygen saturation. When the patient’s critical injuries, like severe oral bleeding and unstable craniofacial fractures, are managed or improved, an awake fiberoptic orotracheal intubation can be performed. Keeping the patient awake allows for better control of spontaneous breathing while dealing with unstable craniofacial structures (Figure 2).

**Figure 3:**
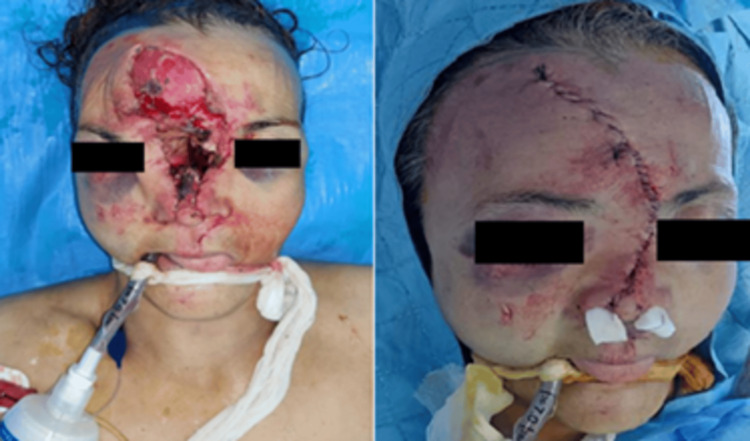
Endotracheal tube introduced via awake bronchoscopy, with significant tissue loss in the frontonasal region, and the immediate postoperative result following debridement, and layered suturing.

We established a rapid-sequence awake fiberoptic oral intubation in several phases. We placed the patient and prepared her in a supine position with a slightly backward-flexed neck and removed obstacles interfering with facial bleeding and restoration of the airway after verifying the absence of cervical spine lesions on the CT scan. A manual cricothyrotomy tray, a two-handed laryngoscope, and an adjustable-height intensive care table were used for this purpose in case of upper airway obstruction during the attempt at orofibroscopic bronchial intubation, given the destruction of the nasal openings, the triangular cartilage, the nasal bone, as well as lesions of the anterior cranial base (the base of the frontal sinus, the cribriform plate, and the frontal sinus bone). After confirming that her airway was free of any blood clots, we sprayed 4% lidocaine through the oral cavity. Three milliliters of 5% lidocaine with naphazoline were administered separately as the most effective topical spray that we have available, along with topical anesthesia of the oral cavity, and we additionally performed bilateral ultrasound-guided superior laryngeal block with an injection of 3 mL of 2% xylocaine on each side, with the patient showing no coughing, no upper airway obstruction, or procedural desaturation. As a precaution, a propofol TCI (target controlled infusion) system was prepared for conscious sedation in case of the patient's non-cooperation, and a percutaneous tracheostomy kit was also ready for use in the intubation trolley. The fiberscope was introduced through a dental spacer placed between the teeth, and orotracheal intubation was performed successfully via fiberoptic guidance after slow progression, with fractional injections of 2% xylocaine into the operating channel through the oropharynx and epiglottitis until below the level of the vocal cords (Figure 3).

After the end of the intervention, the patient was extubated; short-term evolution was favorable; the patient was discharged from the hospital after 10 days of hospitalization with subsequent follow-up showing no particular complications.

## Discussion

Maxillofacial injuries and severe head and neck trauma greatly complicate airway management. The significant and complex nature of these injuries makes it much more challenging to secure and maintain the airway effectively [8]. These challenges may result from various factors, including anatomical abnormalities, inflammation of soft tissues, fractures that are out of place, and bleeding within the mouth. When faced with these situations, standard methods for protecting the airway may not work effectively, leading to the need for more advanced and specialized methods in order to ensure the safety and well-being of the patient. It is essential to address these challenges promptly and efficiently, as they can pose significant risks and complications if left untreated. Therefore, healthcare professionals must continuously stay updated on the latest techniques and technologies to effectively manage and navigate through these complex cases, providing the best possible care and outcomes for their patients. Through thorough assessment, diligent planning, and precise execution, these advanced methods can help alleviate potential obstructions or complications within the airway, ultimately improving patient outcomes and ensuring their overall health and comfort. By embracing innovation and adopting evidence-based practices, healthcare professionals can pave the way for advancements in airway management and enhance the quality of care provided in challenging situations. With a strong interdisciplinary approach and collaboration among medical experts, the development of more specialized techniques can further enhance the ability to successfully overcome airway challenges and provide tailored solutions for each patient's unique needs [9]. One method that can be utilized is fiberoptic intubation, which provides highly precise and detailed visualization of the airway structures, thus ensuring a significantly safer and more controlled intubation process that prioritizes patient safety above all else. This incredibly effective method entails the gentle and careful insertion of a remarkably flexible fiberoptic scope either through the nasal or oral passage, granting the healthcare professional a remarkably clear and crystal-clear view of the delicate larynx and empowering them to skillfully and expertly navigate the placement of the endotracheal tube with utmost precision, efficacy, and finesse, guaranteeing optimal patient outcomes and enhancing overall medical care [10]. Another method that can be utilized in such difficult cases is video laryngoscopy but it requires deep sedation and artificial ventilation, which may not always be feasible or safe, and its effectiveness depends on equipment availability and operator training. This innovative tool provides enhanced visualization, particularly for patients with limited mouth opening or restricted airway access, allowing medical professionals to effectively overcome challenges and ensure successful airway management. Furthermore, in addition to these advanced methods, it is essential to possess a comprehensive understanding of the patient's specific injuries and how they may affect airway management. This involves diligently evaluating the severity of facial fractures, meticulously assessing the stability of the cervical spine, and taking into account any related soft tissue injuries in order to develop a tailored treatment plan. By employing a holistic approach and utilizing cutting-edge technologies, healthcare providers can optimize patient outcomes, providing the utmost quality care in even the most complex cases [11]. In this particular scientific inquiry, a remarkably flexible and versatile fiberoptic bronchoscope was employed to conduct a comprehensive and in-depth examination of the laryngeal inlet. The primary objective of the examination revolved around trauma patients who had sustained substantial craniofacial injuries and required intubation while in a supine position on their backs. This intricate and meticulous investigation aimed to evaluate the condition of the laryngeal inlet in these specific cases, providing valuable insights and crucial information for medical practitioners and researchers alike. By utilizing the advanced capabilities and cutting-edge technology offered by the fiberoptic bronchoscope, this study sought to shed light on the complex challenges faced by healthcare professionals when dealing with intubation procedures in such critical and complex scenarios. Through a systematic and thorough approach, the research team meticulously documented their findings, analyzing a wide range of factors and variables to ensure a comprehensive assessment. Their meticulous examination of the laryngeal inlet allowed for the identification of potential complications and obstacles that arise when intubating trauma patients with significant craniofacial injuries, presenting an opportunity to develop innovative solutions and improve patient care [12]. Regrettably, it was unexpectedly and unfortunately established that visualizing and assessing the laryngeal inlet using the highly advanced method of fiberoptic bronchoscopy presented a notably challenging and complicated obstacle, proving to be quite difficult and arduous, for a rather considerable and significant proportion of the patients who were subject to this procedure. Shockingly, and with great dismay, it was revealed that exactly 23% of these patients encountered this specific impediment, highlighting the magnitude and seriousness of this issue [13]. This unfortunate situation occurred due to simultaneous fractures, trismus, severe bleeding, and unforeseen complications. The medical team quickly acted to address these issues and prevent long-term consequences. They worked meticulously to stabilize fractures, alleviate trismus, control bleeding, and monitor vital signs. Through expertise and resourcefulness, they devised strategies for relief. The team remained dedicated, to managing the patient's condition while prioritizing safety and comfort. After rigorous assessment and care, progress was made. Fractures healed, trismus eased, and bleeding was controlled. The patient's condition stabilized, bringing hope to loved ones. This situation showcases the resilience and dedication of the healthcare team as they navigated this odyssey, ultimately restoring the patient's health [14]. In instances where fiberoptic technique was not available, highly trained medical staff had to use basic methods to perform orotracheal intubation in the supine position to ensure a secure airway. It is crucial to emphasize that when using this alternative approach, patient selection was done with great care, taking into account their individual airway conditions and carefully considering the intubating physician's preferences regarding the presence of bleeding, concurrent fractures, and cervical spine instability [15]. The use of fiberoptic technology is widely regarded as the most effective and reliable method for ensuring precise placement of the endotracheal tube in trauma patients with severe facial fractures, thereby reducing the risk of incorrect placement in the delicate soft tissues of the neck. However, it is important to acknowledge that there may be limitations to the application of the fiberoptic method in trauma patients with facial fractures, primarily stemming from difficulties in visualizing laryngeal structures or a scarcity of proficient medical professionals capable of proficiently executing this intricate technique. As a result, alternative strategies or interventions may need to be considered in these particular cases to ensure the optimal and safe management of the airway in trauma patients [16]. It is important for anesthesiologists to be knowledgeable about the specific challenges involved in managing the airways of these patients. We believe that implementing a standardized protocol for airway management could help reduce complications related to the airway in critical patients with severe craniofacial trauma. If the fiberoptic technique is not available, trained personnel can attempt orotracheal intubation using basic techniques in the supine position, with the administration of lidocaine to suppress the cough reflex. In this case, due to facial trauma, mask ventilation was not feasible, it could worsen the fractures and increase the risk of aspiration. So airway access was achieved through fiberoptic orotracheal intubation. Experience in performing fiberoptic intubation played a crucial role in determining the approach to the airway. The fiberoptic endoscope improves both the speed of intubation and the positioning of the tube, particularly for severely injured patients, and can even be performed by less experienced personnel. Fiberoptic systems have been used to ventilate and oxygenate distal injuries in patients and have also been widely used to facilitate ongoing learning, as the fiberscope enhances understanding of anatomy [17]. Fiberoptic intubation is widely acknowledged as a more challenging procedure than direct laryngoscopy, largely due to the complex geometry and flexibility involved. Our case stands out as an example of a successful primary fiberoptic intubation performed with careful precision, thorough caution, and an established plan that included open surgical airway management to enhance airway safety. Managing craniofacial trauma presents significant challenges and necessitates a multidisciplinary approach. Orotracheal intubation is usually preferred over nasotracheal intubation in patients with severe craniofacial injuries due to the inherent difficulty [18]. This is due to the potential for causing cutting, bleeding, swelling, and alteration of the nasal anatomy. It is essential to prepare for surgery using the Seldinger technique when performing nasotracheal intubation or tracheotomy before administering anesthesia. The associated risks of urgent tracheotomy, narrowing of the area around the vocal cords, and repeated injury to the nerves in the larynx require the exploration of alternative methods for airway access in patients at high risk [19]. Based on our extensive experience and expertise in the field, we firmly believe that fiberoptic orotracheal intubation stands out as the unequivocally superior and supremely secure method for gaining optimal access to the airway in highly intricate cases involving craniofacial trauma. The dynamic interplay and seamless coordination among skilled anesthesiologists, meticulous ICU staff, and proficient surgeons are of paramount importance to ensure the utmost efficacy in providing the necessary oxygenation, ventilation, anesthetic management, and meticulous surgical care for patients grappling with substantial craniofacial trauma. It is absolutely essential to adopt a meticulously planned and diligently executed systematic approach that encompasses all possible contingencies, thereby enabling healthcare professionals to adroitly navigate through complex and potentially perilous scenarios that may arise, ensuring the preservation of life and the delivery of superlative care [20].

## Conclusions

The management of challenging airways in patients with severe craniofacial trauma necessitates a collaborative approach involving skilled anesthesiologists, maxillofacial surgeons, and trauma specialists. Through the application of advanced techniques and a thorough evaluation of the patient's condition, we can enhance airway management and enhance patient outcomes in these demanding circumstances. By working together as a cohesive team, they can effectively address the complexities associated with difficult airway management. The potential complications and risks linked to interventions in patients with severe facial trauma are considerable. Our case illustrates, in a striking manner, that ventilation and orotracheal intubation through classic laryngoscopy are not advisable in severe crush skull fractures. Even fiberoptic intubation with the patient awake becomes unfeasible in cases where occlusion of the jaws impedes the introduction of the epiglottis-retaining action of the intubation instrument. Severe craniofacial trauma poses a challenging situation to resolve in seriously obstructed airways with limited access to the oral cavity. 
